# Association between vitamin D receptor *BsmI* polymorphism and bone mineral density in pediatric patients: A meta-analysis and systematic review of observational studies

**DOI:** 10.1097/MD.0000000000006718

**Published:** 2017-04-28

**Authors:** Li Bao, Mingzhi Chen, Yong Lei, Zemin Zhou, Huiping Shen, Feng Le

**Affiliations:** a Department of Pediatrics; b Department of Thoracic Surgery; c Department of Protection, Affiliated Yixing People Hospital, Jiangsu University, Yixing, China.

**Keywords:** bone mineral density, gene polymorphism, meta-analysis, pediatrics, vitamin D receptor

## Abstract

**Background:**

Vitamin D and the vitamin D receptor (VDR) are important in the metabolic processes that affect bone mineral density (BMD). However, the effect of VDR *BsmI* polymorphism on BMD in pediatric patients is still unclear.

**Methods:**

Eligible studies were identified from the following electronic databases: PubMed, Embase, the Cochrane Library, and the Chinese CNKI and Wanfang databases before October 1, 2016. Data were extracted from the eligible studies, and associations between VDR *BsmI* polymorphism and BMD in pediatric patients were estimated with weighted mean differences (WMDs) and 95% confidence intervals (CIs). Subgroup analysis of ethnicity and sensitivity analyses were used to identify sources of heterogeneity.

**Results:**

A significant difference was observed between VDR *BsmI* polymorphism and pediatric BMD levels of the lumbar spine (LS) in the corecessive model (bb vs BB + Bb: WMD = −0.23, 95% CI [−0.35, −0.11], *P* < 0.01). No significant relationship was found in the dominant, recessive, or codominant models for LS BMD (BB vs Bb: WMD = −0.56, 95% CI [−1.58, 0.46], *P* = 0.29; BB vs bb: WMD = −0.54, 95% CI [−1.49, 0.41], *P* = 0.27; and BB vs Bb + bb: WMD = −0.45, 95% CI [−1.71, 0.26], *P* = 0.22). In addition, we found no remarkable association between the *BsmI* polymorphism and BMD levels of the femoral neck (FN) in children (BB vs Bb: WMD = −1.08, 95% CI [−3.13, 0.96], *P* = 0.30; BB vs bb: WMD = 0.98, 95% CI [−0.89, 2.85], *P* = 0.31; BB vs Bb + bb: WMD = −0.061, 95% CI [−0.30, 0.17], *P* = 0.61; and bb vs BB + Bb: WMD = 0.82, 95% CI [−0.59, 2.32], *P* = 0.25).

**Conclusion:**

Our meta-analysis found that the VDR *BsmI* genetic polymorphism was correlated with LS BMD level in pediatric patients: compared with those with the B allele, children with the bb genotype were less likely to have lower BMD levels. No significant difference was identified in the pediatric FN BMD levels.

## 1 Introduction

Vitamin D plays a vital role in a large variety of metabolic pathways, exerting its actions through the binding of the metabolically active 1,25-dihydroxyvitamin D to the vitamin D receptor (VDR). Therefore, genetic variability in the VDR can have a great impact on the activity and function of vitamin D. The *VDR* gene is located at chromosome 12q13.11 and contains 11 exons and 4 polymorphic regions, namely, *BsmI*, *ApaI*, *FokI*, and *TaqI*. These single-nucleotide polymorphisms (SNPs) have been widely studied and are reported to be strongly associated with autoimmune diseases, such as asthma, multiple sclerosis, and type 1 diabetes.^[1–4]^

Various studies have reported that genetic factors have a great influence on bone mineral density (BMD) levels in both males and females.^[5–7]^ In 1994, Morrison et al^[8]^ proposed that common allelic variants in the *VDR* gene can be used to predict BMD differences and account for up to 75% of the total genetic effect on BMD in healthy individuals. However, the effect of VDR genetic polymorphism, especially in the 4 polymorphic regions, on BMD in children is still uncertain. Therefore, we performed a systematic review and meta-analysis to investigate the association between VDR *BsmI* polymorphism and BMD in children.

## 2 Methods

### 2.1 Ethnic disclosures

The study protocol was in accordance with the ethical standards of the Declarations of Helsinki and Istanbul and was approved by the local Ethics Committee of the Yixing People's Hospital affiliated with Jiangsu University.

### 2.2 Search strategy

Two independent authors (LB and MC) performed comprehensive literature searches in PubMed, the Cochrane Central Register of Controlled Trials, Embase, and the Chinese CNKI and Wanfang databases (updated on October 1, 2016). The following keywords were used: (vitamin D receptor OR VDR) AND (polymorphisms OR SNPs OR variants) AND (children OR child OR pediatric) AND (bone mineral density OR bone mass). The equivalent Chinese terms were used in the Chinese databases. Furthermore, the reference lists of all the studies included in the meta-analysis were reviewed.

### 2.3 Inclusion and exclusion criteria

The inclusion criteria for studies were as follows: case–controlled studies designed to investigate the association between VDR *BsmI* SNP and BMD levels in children, patients from the 3 allelic groups were under 18 years of age, the genotype or allele frequencies and BMD levels for case and control groups were reported, all subjects from the 3 allelic groups were derived from a population within the same geographic area and ethnic background as the controls, and full-text articles published in English or Chinese. Studies with insufficient data for pooling or that did not report the frequency of each polymorphism and outcome were excluded. Two authors (LB and MC) assessed and selected trials for the final analysis independently according to the above criteria, and divergences were subsequently resolved by consensus.

### 2.4 Data extraction

Relevant data from all selected studies were extracted independently by 2 authors (LB and MC). Basic information was collected on each study as follows: first author's name, publication year, study nation, subject number, male/female, mean patient age, and genotyping method. In addition, the results of VDR SNPs and the BMD levels were also collected using a standardized data extraction form. Lastly, missing data were sought by contacting the first or corresponding author.

### 2.5 Statistical analysis

Pooled data were used to assess the strength of the association between VDR polymorphisms and BMD in children by the pooled weighted mean difference (WMD) with 95% confidence intervals (CIs) in a dominant model, a recessive model, a codominant model, a corecessive model, and an allele model. *P* values less than 0.05 were considered statistically significant. Heterogeneity among the trials was determined by *I*^2^, which was defined as 100% × (Q − df)/Q, where Q is Cochran heterogeneity statistic and df is the degrees of freedom, with a fixed-effect model set at low statistical inconsistency (*I*^2^ < 25%). Otherwise, we selected a random-effects model, which is better adapted for clinical and statistical variations.^[9]^ To explore the potential effects of heterogeneity, we carried out stratification analyses by ethnicity, age, and quality criteria. The Egger regression test and the Begg–Mazumdar test based on Kendall tau were used to assess potential publication bias. A cumulative meta-analysis was carried out by the year of publication. All of the statistical analyses were performed using Stata Statistical Software: Release 12 (StataCorp LP; College Station, TX).

## 3 Results

### 3.1 Study characteristics

**Figure 1:**
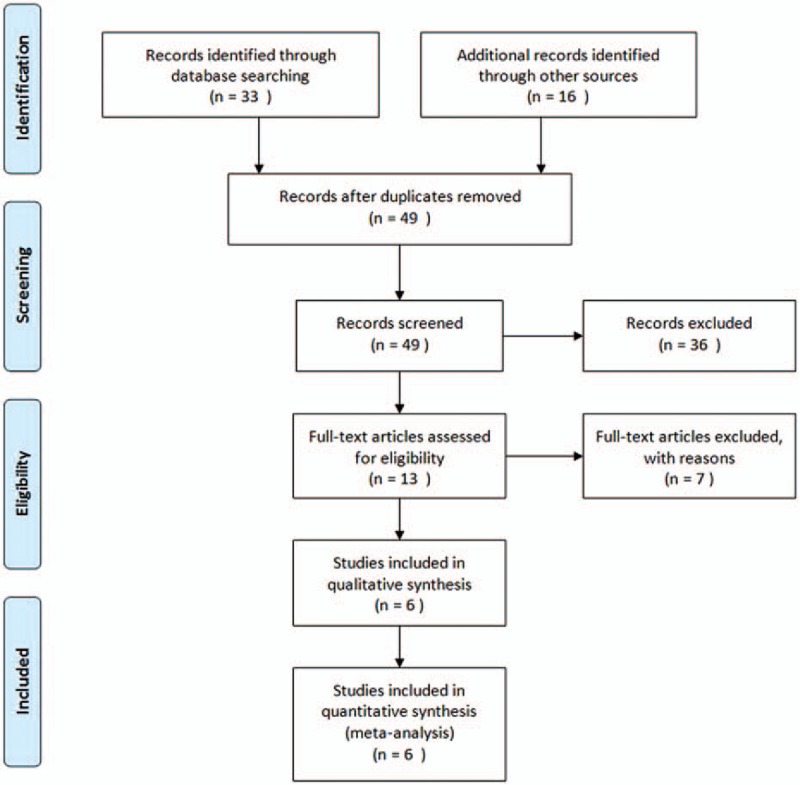
Flow diagram of eligible studies.

A flow diagram of the screening process for the included studies is shown in Fig. 1. Primary screening identified 49 potentially relevant articles, including 33 articles in English and 16 in Chinese. Then, 36 articles were excluded by review of the title or abstract because of the article type or focus or missing data. Screening of the remaining full articles left 6 trials with a total of 1144 pediatric subjects meeting the inclusion criteria for our meta-analysis. Four of the eligible trials studied Caucasian populations, and 2 trials involved Asian populations. Basic characteristics of the eligible studies are shown in Table 1.

**Table 1:**
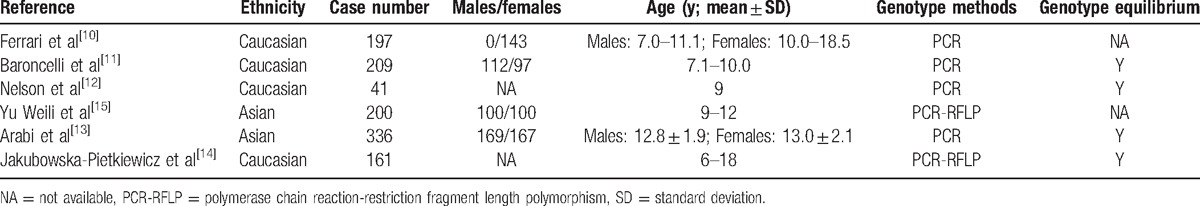
Basic characteristics of subjects in eligible studies.

### 3.2 Quantitative synthesis

**Figure 2:**
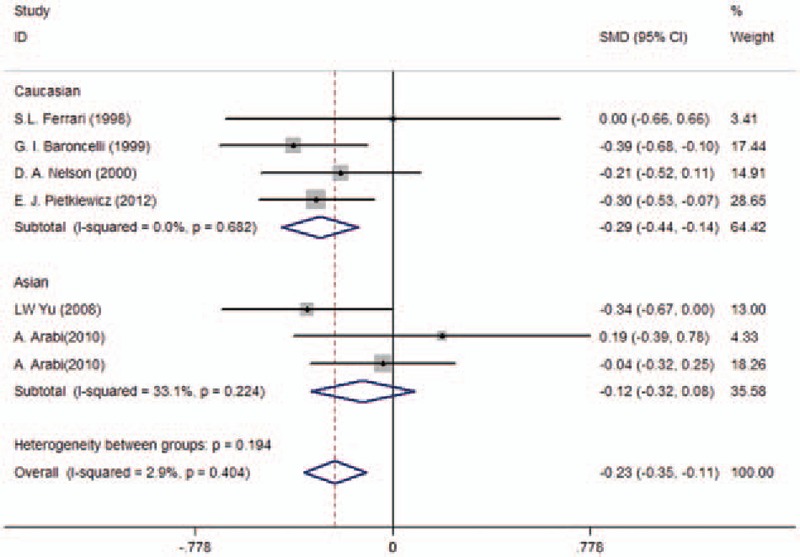
Pooled results of vitamin D receptor *BsmI* genetic polymorphism on pediatric bone mineral density levels in lumbar spine in the corecessive model.

There were 6 trials included in the meta-analysis of the association between VDR *BsmI* polymorphisms and BMD levels in children.^[10–15]^ For BMD levels in the lumbar spine (LS), we observed that *BsmI* genetic polymorphism was significantly associated with BMD levels in the corecessive model (bb vs BB + Bb: WMD = −0.23, 95% CI [−0.35, −0.11], *P* < 0.01; Table 2 and Fig. 2). However, no significant difference was observed in the dominant, recessive, codominant, or allele models (BB vs Bb: WMD = −0.56, 95% CI [−1.58, 0.46], *P* = 0.29; BB vs bb: WMD = −0.54, 95% CI [−1.49, 0.41], *P* = 0.27; BB vs Bb + bb: WMD = −0.45, 95% CI [−1.71, 0.26], *P* = 0.22; Table 2). In addition, we found no significant association in the subgroup analyses for Asian or Caucasian populations. For BMD levels in the femoral neck (FN), there was no significant difference between the *BsmI* polymorphism and BMD levels in children in the dominant, recessive, codominant, or corecessive model (BB vs Bb: WMD = −1.08, 95% CI [−3.13, 0.96], *P* = 0.30; BB vs bb: WMD = 0.98, 95% CI [−0.89, 2.85], *P* = 0.31; BB vs Bb + bb: WMD = −0.061, 95% CI [−0.30, 0.17], *P* = 0.61; bb vs BB + Bb: WMD = 0.82, 95% CI [−0.59, 2.32], *P* = 0.25; Table 2). Similarly, there was no significant association found in the subgroup analysis of ethnicity.

**Table 2:**
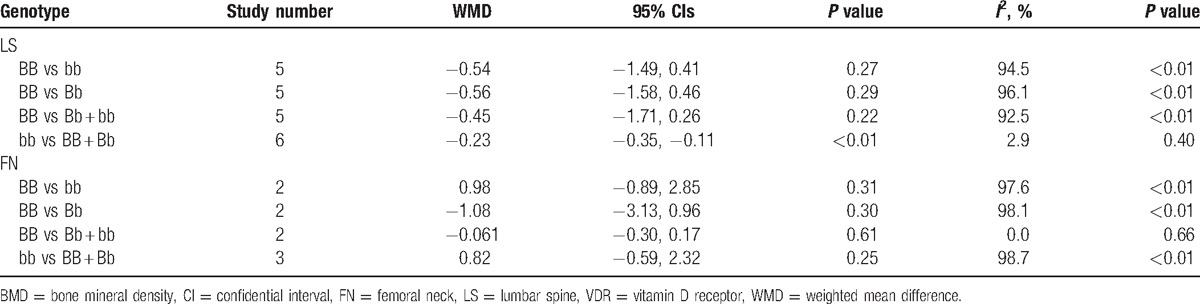
Pooled results of the association between VDR *BsmI* polymorphism and BMD in children.

## 4 Discussion

In this study, we performed a meta-analysis to investigate the association between the VDR *BsmI* polymorphism and BMD levels in children and found that there was a significant correlation between *BsmI* polymorphism and the LS BMD levels in children.

As the active form of vitamin D, 1,25-dihydroxyvitamin D plays a significant role in the metabolism of calcium and influences BMD by binding to the VDR. It has been reported that vitamin D deficiency may contribute remarkably to the loss of BMD and the occurrence of osteoporosis and fracture, especially in older and female patients.^[16,17]^ In 1994, Morrison et al^[8]^ first studied the potential effect of VDR genetic polymorphism on BMD and reported that allelic differences in the 3′ untranslated region of the *VDR* gene may participate in BMD regulation by altering messenger Ribonucleic acid levels. Accordingly, various studies have been performed to further explore the relationship between BMD and VDR SNPs, including the *BsmI* polymorphism. In a 1-year multicenter randomized controlled trial conducted by Palomba et al,^[18]^ a positive correlation was found in postmenopausal osteoporotic women being treated with antiresorptive drugs. Rass et al^[19]^ also found that among patients with rheumatoid arthritis and associated osteoporosis, subjects carrying the B allele showed lower BMD levels and increased bone loss over 1 year, consistent with our findings. Furthermore, it has recently been reported that *BsmI* polymorphism in epilepsy patients taking phenytoin was strongly associated with lower BMD.^[20]^ Similarly, a case–controlled study in Iran suggested a strong association between the VDR *BsmI* polymorphism and LS BMD of Iranian women.^[21]^

However, the effect of *BsmI* polymorphism on BMD is still controversial. A large-scale population-based European study investigated VDR polymorphisms in men and women aged 55 to 80 years and suggested, at most, a small effect of the VDR genotype on BMD in this elderly population.^[22]^ Fang et al^[23]^ performed a meta-analysis of published articles that showed no relationship between the VDR *BsmI* polymorphism and fracture risk, suggesting that this SNP has no impact on BMD. A recent study in Spanish postmenopausal women has also failed to demonstrate an association between the *BsmI* polymorphism and BMD levels.^[24]^ Nevertheless, our meta-analysis showed that children with the B allele of the *BsmI* polymorphism were more likely to have lower LS BMD levels when compared with those with the bb genotype, consistent with the studies showing positive results mentioned above.

Our results should be considered with caution, however, because of certain limitations of our meta-analysis. First, the results were analyzed based on unadjusted estimates. For a more precise analysis, individual data with sufficient information on age, sex, height, weight, lifestyle, and other genetic factors should ideally be used. Second, due to the limited number of studies, we failed to perform subgroup analysis by age and gender. Therefore, the differences between prepubertal and adolescent children and between males and females remain to be determined. Third, only published articles in English and Chinese were included in our analysis, which inevitably resulted in publication and language biases. Limited to our eligible studies, we could not perform an analysis of publication bias, even though all of these issues should be considered in genetic association studies. Besides, considering overwhelming majority of studies in this research were written in English and Chinese, the language bias may be limited.

## 5 Conclusion

In conclusion, our meta-analysis found that the VDR *BsmI* genetic polymorphism was significantly correlated with LS BMD level in pediatric patients, and moreover, compared with those with the B allele, patients with the bb genotype were less likely to have lower BMD levels. No significant association of the VDR *BsmI* polymorphism was found with the FN BMD level in pediatrics. However, larger and more rigorous studies should be conducted to confirm this finding and identify the mechanism by which the VDR *BsmI* polymorphism affects BMD in pediatrics.
